# Supplementary material for: Expression of E-, P- and N-Cadherin and Its Clinical Significance in Cervical Squamous Cell Carcinoma and Precancerous Lesions
Source: PLoS One. 2016 May 25;11(5):e0155910. doi: 10.1371/journal.pone.0155910 (PMC4880319; doi:10.1371/journal.pone.0155910)
Supplement: S1 Table — (DOCX) [file pone.0155910.s001.docx]

**S1 Table. The Correlation of E-P switch or E-N switch with Clinicopathological Characteristics in Patients with Early-stage Cervical Squamous Cell Carcinoma.**

| **Characteristics** | **EP-status,n(%)** | | ***P*** | **EN-status,n(%)** | | ***P*** |
| --- | --- | --- | --- | --- | --- | --- |
|  | **Others** | **E-P switch** |  | **Others** | **E-N switch** |  |
| **Patients age (year)** |  |  | **0.261** |  |  | **0.857** |
| **≤40** | **7(5.5)** | **3(2.4)** |  | **9(7.1)** | **1(0.8)** |  |
| **>40** | **103(81.1)** | **14(11.0)** |  | **113(89.0)** | **4(3.1)** |  |
| **FIGO stage** |  |  | **0.251** |  |  | **0.231** |
| **I** | **83(65.4)** | **10(7.9)** |  | **91(71.7)** | **2(1.6)** |  |
| **II** | **27(21.3)** | **7(5.5)** |  | **31(24.4)** | **3(2.4)** |  |
| **Differentiation** |  |  | **0.702** |  |  | **1.0** |
| **Well/moderate** | **92(72.4)** | **13(10.2)** |  | **101(79.5)** | **4(3.1)** |  |
| **Poor** | **18(14.2)** | **4(3.1)** |  | **21(16.5)** | **1(0.8)** |  |
| **Tumor size(cm)** |  |  | **0.946** |  |  | **0.019** |
| **<4** | **95(74.8)** | **14(11.0)** |  | **107(84.3)** | **2(1.6)** |  |
| **≥4** | **15(11.8)** | **3(2.4)** |  | **15(11.8)** | **3(2.4)** |  |
| **Stromal invasion** |  |  | **0.066** |  |  | **0.234** |
| **<2/3** | **65(51.2)** | **6(4.7)** |  | **70(55.1)** | **1(0.8)** |  |
| **≥2/3** | **45(35.4)** | **11(8.7)** |  | **52(40.9)** | **4(3.1)** |  |
| **Vaginal wall extension** |  |  | **0.573** |  |  | **1.0** |
| **Yes** | **22(17.3)** | **5(3.9)** |  | **26(20.5)** | **1(0.8)** |  |
| **No** | **88(69.3)** | **12(9.4)** |  | **96(75.6)** | **4(3.1)** |  |
| **Parametrial extension** |  |  | **0.092** |  |  | **0.966** |
| **Yes** | **8(6.3)** | **4(3.1)** |  | **11(8.7)** | **1(0.8)** |  |
| **No** | **102(80.3)** | **13(10.2)** |  | **111(87.4)** | **4(3.1)** |  |
| **Endometrial extension** |  |  | **0.003** |  |  | **0.654** |
| **Yes** | **3(2.4)** | **4(3.1)** |  | **6(4.7)** | **1(0.8)** |  |
| **No** | **107(84.3)** | **13(10.2)** |  | **116(91.3)** | **4(3.1)** |  |
| **LVSI** |  |  | **0.027** |  |  | **0.647** |
| **Yes** | **40(31.5)** | **11(8.7)** |  | **48(37.8)** | **3(2.4)** |  |
| **No** | **70(55.1)** | **6(4.7)** |  | **74(58.3)** | **2(1.6)** |  |
| **Surgical margin** |  |  | **0.036** |  |  | **0.857** |
| **Clear** | **104(81.9)** | **13(10.2)** |  | **113(89.0)** | **4(3.1)** |  |
| **Involved** | **6(4.7)** | **4(3.1)** |  | **9(7.1)** | **1(0.8)** |  |
| **LNM** |  |  | **0.001** |  |  | **0.767** |
| **Yes** | **21(16.5)** | **10(7.9)** |  | **29(22.8)** | **2(1.6)** |  |
| **No** | **89(70.1)** | **7(5.5)** |  | **93(73.2)** | **3(2.4)** |  |

**FIGO, International Federation of Gynecology and Obstetrics; LVSI, lymph vascular space invasion; LNM, lymph node metastasis.**
