# Supplementary material for: Expression of E-, P- and N-Cadherin and Its Clinical Significance in Cervical Squamous Cell Carcinoma and Precancerous Lesions
Source: PLoS One. 2016 May 25;11(5):e0155910. doi: 10.1371/journal.pone.0155910 (PMC4880319; doi:10.1371/journal.pone.0155910)
Supplement: S1 Text — (PDF) [file pone.0155910.s002.pdf]

International Science Editing

www.internationalscienceediting.com

Compuscript Ltd
T/A International Science Editing
Bay K, Shannon Industrial Park West
Shannon, Co Clare
Ireland
Tel. +353 61 472818 Fax +353 61 472688

DATE: April 11, 2016

To whom it may concern,

The paper "Expression of E-, P- and N-Cadherin and its Clinical Significance in Cervical Squamous Cell Carcinoma and Precancerous Lesions" by Baohua Li was edited by International Science Editing. We were asked not to check the acknowledgements, references and tables. Please contact us if you would like to view the edited paper.

Kindest regards,

David Cushley.
