# Supplementary material for: Expression of E-, P- and N-Cadherin and Its Clinical Significance in Cervical Squamous Cell Carcinoma and Precancerous Lesions
Source: PLoS One. 2016 May 25;11(5):e0155910. doi: 10.1371/journal.pone.0155910 (PMC4880319; doi:10.1371/journal.pone.0155910)
Supplement: S2 Text — (DOC) [file pone.0155910.s003.doc]

**Expression of E-, P- and N-cadherin and Its Clinical Significance in Cervical Squamous Cell Carcinoma and Precancerous Lesions**

**Abstract**

Aberrant expression of classical cadherins has been observed in tumor invasion and metastasis, but its involvement in cervical carcinogenesis and cancer progression is not clear. We investigated E-, P- and N-cadherin expression and its significance in cervical squamous cell carcinoma (SCC) and cervical intraepithelial neoplasia (CIN). This retrospective study enrolled 508 patients admitted to Women's Hospital, School of Medicine, Zhejiang University with cervical lesions between January 2006 and December 2010. Immunochemical staining was performed in 98 samples of normal cervical epithelium (NC), 283 of CIN, and 127 of early-stage SCC. The association of cadherin staining with clinical characteristics and survival of the patients was evaluated by univariate and multivariate analysis. We found gradients of decreasing E-cadherin expression and increasing P-cadherin expression from NC through CIN to SCC. Aberrant E-cadherin and P-cadherin expression were significantly associated with clinical parameters indicating poor prognosis and shorter patient survival. Interestingly, we found very low levels of positive N-cadherin expression in CIN and SCC tissues that were not related to CIN or cancer. Pearson chi-square tests showed that E-cadherin expression in SCC was inversely correlated with P-cadherin expression (E-P switch), and was not correlated with N-cadherin expression. More important, patients with tissues exhibiting an E-P switch in expression had highly aggressive phenotypes and poorer prognosis than those without E-P switch expression. Our findings suggest that E-cadherin and P-cadherin, but not N-cadherin staining, might be useful in diagnosing CIN and for predicting prognosis in patients with early-stage SCC.

**Key words: cervical squamous cell carcinoma; cervical intraepithelial neoplasia; E-cadherin; P-cadherin; N-cadherin**

**Introduction**

Cervical cancer is one of the most common malignant gynecological tumors, with an estimated 527,600 new cases and 265,700 deaths worldwide in 2012 [1]. In recent decades, the incidence rate of cervical cancer in developed countries has gradually declined due to the widespread use of cervical screening. In less developed countries including China, cervical cancer remains the second most commonly diagnosed cancer and the third leading cause of cancer death in women [1].

Cervical carcinogenesis is induced by persistent high-risk HPV infection and progresses slowly. The cervical epithelium transforms from normal tissue through cervical intraepithelial neoplasia (CIN) to cervical cancer. CIN can be characterized as a low-grade lesion (LSIL) or high-grade lesion (HSIL). Most LSILs regress spontaneously, whereas the majority of HSILs progress to invasive disease [2]. However, there is a current lack of biomarkers to predict progression to cervical cancer in women with CIN.

Radical surgery is a first-choice treatment for early stage cervical cancer patients. Patients with poor prognosis should receive radiation or chemo-radiation after surgery. Lymph node metastasis (LNM) has been recognized as an indicator of poor prognosis and an independent variable associated with poor survival [3,4]. Previous studies have reported that the 5-year survival rate of patients with LNM was 20% to 30 % lower than that of patients without LNM [5]. A better understanding of the mechanisms of cervical cancer metastasis and identification of therapeutic targets associated with metastasis, would be expected to contribute to improving the prognosis of cervical cancer patients.

The cadherin superfamily is an evolutionarily conserved family of adhesion molecules that mediate calcium-dependent, homophilic cell-cell interactions. Cadherin molecules can be subdivided into three subfamilies: classical cadherins, nonclassical cadherins and protocadherins. Of these, classical cadherins are the most extensively studied. Based on their tissue distribution, classical cadherins comprise mainly epithelial cadherin (E-cadherin), placental cadherin (P-cadherin), and neural cadherin (N-cadherin) [6,7], which are all required for normal cell-cell adhesion and maintaining tissue integrity and homeostasis. Aberrant expression of classical cadherins was recently found to be associated with tumor invasion and worse prognosis in many carcinomas [8,9]. E-cadherin has been identified as a tumor suppressor gene [10,11], and is recognized as having a role in the development of cervical cancer. Decreased E-cadherin expression has not only been detected in cervical squamous carcinoma [12,13], but also has been associated with tumor invasion and poor prognosis [14]. Several regulatory factors, including transforming growth factor β1 [15], KCL cotransporter-3 [16], and epidermal growth factor [17], promote cell migration and invasion in cervical cancer cell lines by regulating the expression and function of E-cadherin. However, E-cadherin expression in CIN and its role in cervical carcinogenesis have not been described. In addition, few if any data on the expression and function of the other two family members, P-cadherin and N-cadherin, in cervical squamous cell cancer (SCC) and its precursors are available.

We examined the expression of E-, P- and N-cadherin in tissues from normal cervical squamous epithelium, CIN and early-stage cervical squamous carcinoma by immunohistochemistry. The aim of this study was to describe the level of expression of the cadherins and their clinical significance in early stage SCC and its precursors.

**Materials and Methods**

**Ethics statement**

The present study was approved by the Institutional Review Board, Women’s hospital, School of Medicine, Zhejiang University. The informed consent has been given and obtained from all individual participants included in the study.

**Patients and tissue specimens**

This retrospective study enrolled 508 patients with cervical lesions treated at Women’s Hospital, School of Medicine, Zhejiang University between January 2006 and December 2010. The study population comprised two groups. One included 127 patients with early stage SCC who underwent radical hysterectomy with pelvic lymph node dissection and did not receive any anticancer therapy prior to their surgery. The clininicopathological characteristics of those patients are shown in Table.2. The second group included 283 CIN patients with colposcopy and biopsy at the Hospital’s cervical lesion clinic because of abnormal cytology (liquid-based cytological test) and/or high-risk HPV positive (HC2 method). Normal cervical epithelial tissue was collected from 98 control patients with hysterectomies due to benign gynecologic diseases.

**Follow-up**

The patients with SCC were followed up postoperatively by interview at the clinic or by phone call. Regional tumor recurrence, distant metastasis, and patient survival were recorded; disease-free survival (DFS) and overall survival (OS) were calculated from the day of the surgery until recurrence or death. The last day of follow-up was December 2014. The median follow-up was 72 months (range, 13–99 months). During follow-up, 21 patients (16.5 %) had a clinical recurrence and 16 (12.6 %) died of progressive disease; 12 were lost to follow up.

**Immunohistochemical staining**

Tissues were embedded in paraffin, sectioned at 4μm, mounted on slides, dewaxed in xylene for 15 minutes, passed through a graded aqueous/alcohol series, and rehydrated in distilled water. To recover antigen reactivity, sections were heated in 0.1 mol/L pH 6 citrate buffer for 20 min in a 95 °C water bath, cooled, and kept at room temperature for 30 min. Nonspecific binding was blocked by incubating the sections for 30 min with diluted normal goat serum. The immunohistochemical staining was performed by the avidin-biotin peroxidase method using a SLABC kit (DAKO, Glostrup, Denmark). Briefly, slides were incubated at room temperature for 2 h with polyclonal rabbit E-cadherin antibody (1:500; Catalog Number, sc-7870; Santa Cruz, CA, USA), polyclonal rabbit P-cadherin antibody (1:200; Catalog Number, sc-7893; Santa Cruz), or polyclonal rabbit N-cadherin antibody (1:50; Catalog Number, sc-7939; Santa Cruz). Then the sections were developed for 2 min with the enzyme substrate 3,3’-diaminobenzidine chromagen (DAB, DAKO) and counterstained with Mayer’s hematoxylin for 30 s. After dehydration, the tissues were coverslipped with Permount and observed.

**Evaluation of immunoreactivity**

Immunohistochemical staining was independently scored in four fields by two senior pathologists who had no knowledge of the clininicopathological data. In case of disagreement, the scoring was discussed by the pathologists until they agreed on a final result. E-cadherin and P-cadherin immunoreactivity was assessed by staining intensity and the distribution of positively stained tumor cells. Intensity was scored as negative (0), weakly positive (1), or moderately or strongly positive (2). The staining distribution was scored as 0 for <10 %, 1 for 10 to <50 %, and 2 for ≥50 % positively stained cells. The sum of the staining intensity and distribution scores was used to express immunoreactivity. A total score <3 was considered as reduced expression and ≥3 as increased expression. As previously reported, N-cadherin expression was considered positive if at least 5% of the cells had moderate or strong membrane and/or cytoplasmic staining [18,19]. Tissue samples with fewer or less intensely stained cells were scored as negative.

**Statistical analysis**

Statistical analyses were performed using SPSS 16.0 for windows (SSPS Inc, Chicago, IL, USA). The correlation of cadherin expression with clinicopathological characteristics, and the significance of differences in cadherin expression were determined by Pearson’s chi-square test. Survival curves were generated by the Kaplan–Meyer method and survival rates were compared using the log-rank test. On the univariate and multivariate analyses, the Cox proportional hazard method was used to identify independent predictors of survival. All statistical tests were two-sided, and *P*-values ≤0.05 were considered statistically significant.

**Results**

**The evaluation of E-, P- and N-cadherin expression in cervical specimens**

A total of 508 cervical tissue samples were collected to evaluate E-, P- and N-cadherin expression by immunohistochemistry, including 98 NC, 283 CIN, and 127 early stages SCC. The mean patient age was 39.40 years for NC, 39.98 for CIN, and 50.18 for SCC. Among patients with CIN, 74 had an LSIL and 209 had an HSIL. Of the patients with early stage cervical cancer, 93 were Stage I and 34 were Stage II (International Federation of Obstetrics and Gynecology, 2000); 4 cases were well differentiated (G1), 101 were moderately differentiated (G2), and 22 were poorly differentiated (G3). Lymph node metastasis (LNM) was confirmed in 31 (24.41 %) of 127 cases. Immunohistochemical staining of E-, P- and N-cadherin expression is shown in Figure 1 and described below.

**E-cadherin expression**

The majority of NC epithelium tissue samples showed consistent, strong staining of E-cadherin of cell membranous throughout all layers. E-cadherin immunoreactivity was also observed predominantly in the cell membrane of cervical cancer cells, whereas stromal cells did not exhibit any staining. As shown in Table 1, 97.96% (96/98) samples of NC epithelium had increased expression of E-cadherin (scores ≥3) and only 2.04% had reduced expression (scores <3). However, reduced expression of E-cadherin was seen in 10.25% (29/283) CIN specimens and 35 of 127 SCC specimens (27.56%). E-cadherin expression gradually decreased from the level seen in normal epithelium through CIN to SCC (NC vs. CIN, *P*=0.01; NC vs. SCC, *P*=3.05E-7; CIN vs. SCC, *P*=7.98E-6). Also, in CIN, E-cadherin expression was significantly lower in HSIL than in LSIL and in normal epithelium (HSIL vs. LSIL, *P*=0.041; HSIL vs. NC, *P*=0.003). E-cadherin expression in LSIL and NC were not significantly different (NC vs. LSIL, *P*=0.749).

**P-cadherin expression**

P-cadherin immunostaining was absent or very weak in normal cervix, and predominantly restricted to the basal one or two layers of the epithelium. In SCC, P-cadherin was predominantly localized in the membrane and cytoplasm of tumor cells without significant nuclear staining. As shown in Table 1, P-cadherin expression increased with progression from NC through CIN to SCC. Increased expression of P-cadherin (scores ≥3) was observed in 29 of 283 CIN (10.25%) and 41 of 127 SCC (32.28%) specimens compared with 2/98 NC patients (2.04%); all differences were significant (NC vs. CIN, *P*=0.01; NC vs. SCC, *P*=1.06E-8; CIN vs. SCC, *P*=4.17E-8). P-cadherin expression was significantly higher in both HSIL and LSIL than that in NC tissue (HSIL vs. NC, *P*=0.03; LSIL vs. NC, *P*=0.002), but expression of P-cadherin in HSIL and LSIL did not differ significantly (*P*=0.127).

**N-cadherin expression**

As shown in Table 1, there was no detectable N-cadherin staining in NC epithelium (0/98). In SCC, N-cadherin staining was predominantly located in the membrane and cytoplasm of tumor cells. Positive N-cadherin staining was seen in 12 of 283 CIN (4.24%) and 16 of 127 SCC (12.59%), with a progressive increase in N-cadherin expression along with the progression of cervical disease (NC vs. CIN, *P*=0.083; NC vs. SCC; *P*=2.66E-4; CIN vs. SCC, *P*=0.002). N-cadherin expression in NC, LSIL, and HSIL did not differ significantly (LSIL vs. NC, *P*=0.031; HSIL vs. NC, *P*=0.155; HSIL vs. LSIL; *P*=0.360).

**The correlation of E-, P- and N-cadherin expression with clinicopathological characteristics in SCC**

The correlation of E-, P-, and N-cadherin expression with clinicopathological characteristics of SCC tissues is shown in Table 2. Reduced E-cadherin staining (score <3) was seen in tissue from 35 early stage SCC patients (27.56%), and increased staining (score ≥3) was seen in the remaining 92 samples (72.44%). Reduced E-cadherin expression was significantly associated with lymphovascular space invasion (LVSI) (*P*=0.045) and lymph node metastasis (LNM) (*P*=0.001). P-cadherin expression was reduced (score <3) in 86 (67.72%) specimens and increased (score ≥3) in 41 (32.28%). Increased P-cadherin expression was significantly associated with the degree of stromal invasion (*P*=0.024), LVSI (*P*=0.011), and LNM (*P*=0.002). N-cadherin expression was negative in 111 (87.40%) specimens and positive in 16 (12.60%). Positive N-cadherin expression was correlated only with the degree of stromal invasion (*P*=0.034).

**The correlation of P-cadherin or N-cadherin with E-cadherin expression in SCC**

The Pearson chi-square test showed that E-cadherin expression was inversely correlated with P-cadherin expression (*P*=0.015), but not correlated with N-cadherin expression (*P*=0.957), as shown in Table 3. The results suggest that there is an E-P switch, but not an E-N switch, in early-stage SCC tissue.

**The correlation of E-P switch with clinicopathological characteristics in SCC**

Simultaneous down-regulation of E-cadherin expression and up-regulation of P-cadherin expression (i.e., an E-P switch) was seen in 17 (13.39%) early-stage SCC patients. The relationship of the cadherin switch profile with clinicopathological characteristics is shown in Figure 2. Some differences in the clinicopathological characteristics of patients with or without E-P switch profiles were significant. As shown in Table S1, these included endometrial extension (*P*=0.003), LVSI (*P*=0.027), surgical margin (*P*=0.036), and LNM (*P*=0.001).

**Survival analysis**

Kaplan–Meier analysis was used to investigate the prognostic value of the expression of classical cadherins. The survival curves in Figure 3 show the association of E-, P- and N-cadherin expression with disease-free survival (DFS) and overall survival (OS) in 127 patients with early-stage SCC. Significance was tested in univariate and multivariate Cox regression models. The Kaplan–Meier analysis showed that reduced E-cadherin expression was significantly associated with shorter DFS (*P*=3.89E-5) and OS (*P*=0.001). Patients with increased P-cadherin expression also had significantly shorter DFS (*P*=0.028) and OS (*P*=0.008), N-cadherin expression was not significantly associated with DFS (*P*=0.779) or OS (*P*=0.550). Kaplan-Meier analysis also showed that the E-P switch was strongly associated with shorter DFS (*P*=0.001) and OS (*P*=0.002) compared with survival in the absence of the E-P switch. Furthermore, Cox univariate proportional hazards analysis showed that International Federation of Gynecology and Obstetrics (FIGO) stage, the degree of stromal invasion, parametrial extension, endometrial extension, LVSI, surgical margin, LNM, E-cadherin expression, P-cadherin expression, and E-P switch profile predicted significantly shorter DFS and OS. More important, multivariate analysis showed that both reduced E-cadherin expression and LNM, were independent predictors of shorter DFS and OS in patients with early stage SCC (Table 4).

**Discussion**

Classical cadherins, the transmembrane components of adherens junctions, bind to cytoplasmic catenin molecules and then interact with the actin cytoskeleton. They play a key role in embryogenesis and maintenance of normal adult tissue architecture and cell polarity [20]. E-cadherin is a classical cadherin found in almost all human epithelial tissues, where it mediates the integrity of cell groups and tissue homeostasis in normal epithelia. P-cadherin is predominantly expressed in placental tissue, and is expressed only in the basal layers of stratified epithelium. N-cadherin occurs predominantly in neural tissues, but is usually absent from normal epithelia [21]. A number of studies have demonstrated that significant changes in the expression or function of these classical cadherins can cause abnormal adherens junctions, eventually contributing to initiation and development of malignancy [22,23]. The way in which these classical cadherins are involved in carcinogenesis and cancer progression in the cervix has not been described.

In this study, we found reduced E-cadherin expression and increased P-cadherin expression in patients with LSIL and in those with HISL, suggesting that aberrant expression of both cadherins occurred in the early phase of cervical squamous carcinogenesis. Previous studies have reported similar results [12,24]. It is clear that LSIL and HSIL are different clinical stages of cervical carcinogenesis. One study reported that more than 60 % of LSILs spontaneously regressed within 2 years [25], but that most HSILs progressed to invasive disease if not treated. Based on American Academy of Vaginal and Cervical pathology (ASCCP) guidelines, patients with HSIL require prompt treatment, but that patients with LSILs—with ASC-US or LSIL cytology—should be followed up carefully. Therefore, to choose the appropriate therapeutic strategy, it is important to correctly distinguish between HSIL and LSIL. Our study is consistent with others, and adds to the evidence that E-cadherin and P-cadherin are useful biomarkers, and that immunostaining can assist in distinguishing HSIL from LSIL.

Previous studies also showed that aberrant expression of E-cadherin might be involved in cervical cancer metastasis. Cheng et al reported that decreased expression of E-cadherin and increased expression of vimentin contributed to invasiveness and poor prognosis in cervical cancer [14]. The association of P-cadhern expression with progression of cervical cancer is poorly understood. In this study, we found that decreased E-cadherin expression and increased P-cadherin expression were significantly associated with some clinical variables (e.g., LVSI and LNM) that indicated poor prognosis. The survival analysis showed that reduced E-cadherin expression and increased P-cadherin expression were significantly associated with shorter DFS and OS. Multivariate analysis further verified that E-cadherin expression, like lymph node metastasis (LNM), was an independent predictor of shorter DFS (HR=0.117, 95%CI=0.022-0.619) and OS (HR=0.096, 95%CI=0.012-0.790) of early-stage SCC patients. Thus, our results suggest that at least E-cadherin might be a key biomarker of poor prognosis of early-stage SCC.

The cadherin switch, which is a key characteristic of epithelial-mesenchymal transition (EMT), consists of changes in the expression of different isoforms of classical cadherins that result in alteration of adherens junctions [26]. There is accumulating evidence that the cadherin switch is involved in diverse biological processes including cell adhesion, cell function, and growth regulation. Recent studies have also reported that the cadherin switch was associated with cancer initiation, invasion, and metastasis [27,28]. In this study, we found that E-cadherin expression and P-cadherin expression was inversely correlated (E-P switch) in 127 patients with early-stage SCC. We also observed that SCC expressing the E-P switch had highly aggressive phenotypes and had a poorer prognosis compared with tumors lacking the E-P switch. Theoretically, the predictive value of the E-P switch is greater than that of either E- or P-cadherin alone, but our results did not find that the E-P switch was an independent predictor of patient survival. The reason may be related to the absence of advanced stage patients in our series.

N-cadherin is a classical cadherin with aberrant expression in various human malignancies [29,30], but we found very low levels of N-cadherin expression in cervical lesions and that N-cadherin expression was not associated with clininicopathological characteristics or patient survival in early stage SCC. Thus, N-cadherin did not predict SCC metastasis or prognosis in SCC patients.

**Conclusion**

We showed, for the first time, gradually decreasing E-cadherin expression and gradually increasing P-cadherin expression beginning in NC epithelium and continuing through CIN to SCC. Aberrant E-cadherin and P-cadherin expression or an E-P switch was significantly associated with some clinical variables indicative of poor prognosis and survival in early-stage SCC patients. Our findings suggest that E-cadherin and P-cadherin immunostaining might assist in the diagnosis of CIN and indicating prognosis in early-stage SCC patients.

**Acknowledgements**

We thank Caiyun Zhou for the guidance and implementation of the experiments.

**Author Contributions**

Conceived and designed the experiments: LiBH ChengXD. Performed the experiments: LiBH ShiHY WangFF HongD. Analyzed the data: LiBH ShiHY LvWG XieX ChengXD. Contributed reagents/materials/analysis/tools: LiBH ShiHY LvWG XieX ChengXD. Wrote the paper: LiBH LvWG XieX ChengXD.

References

1. Lindsey A. Torre, Freddie Bray, Rebecca L. Siegel, Jacques Ferlay, Joannie Lortet-Tieulent, Ahmedin Jemal. Global Cancer Statistics, 2012. CA Cancer J Clin. 2015;65:87-108.
2. Holowaty P, Miller AB, Rohan T, To T. natural history of dysplasia of the uterine cervix. J Natl Cancer Inst. 1999;91:1420A-1421.
3. Landoni F, Maneo A, Colombo A, Placa F, Milani R, Perego P, et al. Randomised study of radical surgery versus radiotherapy for stage Ib-IIa cervical cancer. Lancet. 1997;350:535-40.
4. Biewenga P, van der Velden J, Mol BW, Stalpers LJ, Schilthuis MS, et al. Prognostic model for survival in patients with early stage cervical cancer. Cancer. 2011;117:768-76.
5. Shingleton HM, Jones WB, Russell A, Fremgen A, Chmiel JS, Ocwieja K, et al. Hysterectomy in invasive cervical cancer: a national patterns of care study of the American College of Surgeons. J Am Coll Surg. 1996;183:393-400.
6. Lilien J, Balsamo J, Arregui C, Xu G. Turn-off, drop-out: functional state switching of cadherins. Dev Dyn. 2002;224:18-29.
7. Hong S, Troyanovsky RB, Troyanovsky SM. Cadherin exits the junction by switching its adhesive bond. J Cell Biol. 2011;192:1073-83.
8. Gama A, Schmitt F. Cadherin cell adhesion system in canine mammary cancer: a review. Vet Med Int. 2012;2012:357187.
9. Paredes J, Figueiredo J, Albergaria A, Oliveira P, Carvalho J, Ribeiro AS, et al. Epithelial E- and P-cadherins: role and clinical significance in cancer. Biochim Biophys Acta. 2012;1826:297-311.
10. Al-Aynati MM, Radulovich N, Riddell RH, Tsao MS. Epithelial-cadherin and beta-catenin expression changes in pancreatic intraepithelial neoplasia. Clin Cancer Res. 2004;10:1235-40.
11. Muller S, Su L, Tighiouart M, Saba N, Zhang H, Shin DM, et al. Distinctive E-cadherin and epidermal growth factor receptor expression in metastatic and nonmetastatic head and neck squamous cell carcinoma: predictive and prognostic correlation. Cancer. 2008;113:97-107.
12. Rodríguez-Sastre MA, González-Maya L, Delgado R, Lizano M, Tsubaki G, Mohar A, et al. Abnormal distribution of E-cadherin and beta-catenin in different histologic types of cancer of the uterine cervix. Gynecol Oncol. 2005;97:330-6.
13. Koay MH, Crook M, Stewart CJ. Cyclin D1, E-cadherin and beta-catenin expression in FIGO Stage IA cervical squamous carcinoma: diagnostic value and evidence for epithelial-mesenchymal transition. Histopathology. 2012;61:1125-33.
14. Cheng Y, Zhou Y, Jiang W, Yang X, Zhu J, Feng D, et al. Significance of E-cadherin, β-catenin, and vimentin expression as postoperative prognosis indicators in cervical squamous cell carcinoma. Hum Pathol. 2012;43:1213-20.
15. Yi JY, Hur KC, Lee E, Jin YJ, Arteaga CL, Son YS. TGFbeta1 -mediated epithelial to mesenchymal transition is accompanied by invasion in the SiHa cell line. Eur J Cell Biol. 2002;81:457-68.
16. Hsu YM, Chen YF, Chou CY, Tang MJ, Chen JH, Wilkins RJ, et al. KCl cotransporter-3 down-regulates E-cadherin/beta-catenin complex to promote epithelial-mesenchymal transition. Cancer Res. 2007;67:11064-73.
17. Lee MY, Chou CY, Tang MJ, Shen MR. Epithelial-mesenchymal transition in cervical cancer: correlation with tumor progression, epidermal growth factor receptor overexpression, and snail up-regulation. Clin Cancer Res. 2008;14:4743-50.
18. Pyo SW, Hashimoto M, Kim YS, Kim CH, Lee SH, Johnson KR, et al. Expression of E-cadherin, P-cadherin and N-cadherin in oral squamous cell carcinoma: correlation with the clinicopathologic features and patient outcome. J Craniomaxillofac Surg. 2007;35:1-9.
19. Lade-Keller J, Riber-Hansen R, Guldberg P, Schmidt H, Hamilton-Dutoit SJ, Steiniche T. E- to N-cadherin switch in melanoma is associated with decreased expression of phosphatase and tensin homolog and cancer progression. Br J Dermatol. 2013;169:618-28.
20. Jeanes A, Gottardi CJ, Yap AS. Cadherins and cancer: how does cadherin dysfunction promote tumor progression? Oncogene. 2008;27:6920-9.
21. Stemmler MP. Cadherins in development and cancer. Mol Biosyst. 2008;4:835-50.
22. Lascombe I, Clairotte A, Fauconnet S, Bernardini S, Wallerand H, Kantelip B, et al. N-cadherin as a novel prognostic marker of progression in superficial urothelial tumors. Clin Cancer Res. 2006;12:2780-7.
23. Usui A1, Ko SY, Barengo N, Naora H. P-cadherin promotes ovarian cancer dissemination through tumor cell aggregation and tumor-peritoneum interactions. Mol Cancer Res. 2014;12:504-13.
24. de Boer CJ, van Dorst E, van Krieken H, Jansen-van Rhijn CM, Warnaar SO, Fleuren GJ, et al. Changing roles of cadherins and catenins during progression of squamous intraepithelial lesions in the uterine cervix. Am J Pathol. 1999;155:505-15.
25. Rouzier R. Management of CIN1. J Gynecol Obstet Biol Reprod (Paris). 2008;37 Suppl 1:S114-20.
26. Wheelock MJ, Shintani Y, Maeda M, Fukumoto Y, Johnson KR. Cadherin switching. J Cell Sci. 2008;121:727-35.
27. Sun L, Hu H, Peng L, Zhou Z, Zhao X, Pan J, et al. P-cadherin promotes liver metastasis and is associated with poor prognosis in colon cancer. Am J Pathol. 2011;179:380-90.
28. Gravdal K, Halvorsen OJ, Haukaas SA, Akslen LA. A switch from E-cadherin to N-cadherin expression indicates epithelial to mesenchymal transition and is of strong and independent importance for the progress of prostate cancer. Clin Cancer Res. 2007;13:7003-11.
29. Hui L, Zhang S, Dong X, Tian D, Cui Z, Qiu X. Prognostic significance of twist and N-cadherin expression in NSCLC. PLoS One. 2013;8:e62171.
30. Chung S, Yao J, Suyama K, Bajaj S, Qian X, Loudig OD, et al. N-cadherin regulates mammary tumor cell migration through Akt3 suppression. Oncogene. 2013;32:422-30.

**FIGURE LEGENDS**

**Figure.1** –Representative immunohistochemical staining showing E-, P- and N-cadherin expression in normal cervical (NC) epithelium, cervical intraepithelial neoplasia (CIN) and cervical squamous cell carcinoma (SCC) tissue. Strong membranous staining of E-cadherin in all layers of normal cervical epithelium (A). Reduced or increased expression of E-cadherin in low-grade CIN (B, C), high-grade CIN (D, E) and SCC (F, G). Weak membranous and/or cytoplasmic P-cadherin staining in one or two basal layers of NC epithelium (H). Reduced or increased P-cadherin expression in low-grade CIN (I, J), high-grade CIN (K, L) and SCC (M, N). No detectable staining of N-cadherin in NC epithelium (O). Very low rates of positive N-cadherin expression in CIN (P, Q) and SCC (R). Magnifications, ×200.

**Figure.2** –Correlation of the E-P switch and several clinical variables. Among 127 early-stage SCC patients, 17 presented with simultaneously reduced E-cadherin expression and increased P-cadherin expression (an E-P switch). The E-P switch was significantly associated with endometrial extension (*P*=0.003), lymph vascular space invasion (LVSI) (*P*=0.027), surgical margin (*P*=0.036), and lymph node metastasis (LNM) (*P*=0.001).

**Figure.3** –Kaplan–Meyer curves showing the association of aberrant expression of E-cadherin, P-cadherin, N-cadherin, and the E-P switch with patient disease-free survival (DFS) and overall survival (OS). Reduced E-cadherin (A) and increased P-cadherin expression (B), as well as the E-P switch (D), were significantly associated with shorter DFS and OS. N-cadherin expression was not significantly associated with DFS or OS (C).
